# A Large Deletion in the *NSDHL* Gene in Labrador Retrievers with a Congenital Cornification Disorder

**DOI:** 10.1534/g3.117.1124

**Published:** 2017-07-21

**Authors:** Anina Bauer, Michela De Lucia, Vidhya Jagannathan, Giorgia Mezzalira, Margret L. Casal, Monika M. Welle, Tosso Leeb

**Affiliations:** * Institute of Genetics, Vetsuisse Faculty, University of Bern, 3001, Switzerland; † Dermfocus, Vetsuisse Faculty, University of Bern, 3001, Switzerland; ‡ San Marco Veterinary Clinic and Laboratory, Padova 35141, Italy; § Section of Medical Genetics, Department of Clinical Studies, School of Veterinary Medicine, University of Pennsylvania, Philadelphia, Pennsylvania 19104; ** Institute of Animal Pathology, Vetsuisse Faculty, University of Bern, 3001, Switzerland

**Keywords:** dog, canis lupus familiaris, X-chromosome, dermatology, skin, lines of Blaschko, whole genome sequencing, animal model

## Abstract

In heterozygous females affected by an X-linked skin disorder, lesions often appear in a characteristic pattern, the so-called Blaschko’s lines. We investigated a female Labrador Retriever and her crossbred daughter, which both showed similar clinical lesions that followed Blaschko’s lines. The two male littermates of the affected daughter had died at birth, suggesting a monogenic X-chromosomal semidominant mode of inheritance. Whole genome sequencing of the affected daughter, and subsequent automated variant filtering with respect to 188 nonaffected control dogs of different breeds, revealed 332 hetero-zygous variants on the X-chromosome private to the affected dog. None of these variants was protein-changing. By visual inspection of candidate genes located on the X-chromosome, we identified a large deletion in the *NSDHL* gene, encoding NAD(P) dependent steroid dehydrogenase-like, a 3β-hydroxysteroid dehydrogenase involved in cholesterol biosynthesis. The deletion spanned >14 kb, and included the last three exons of the *NSDHL* gene. By PCR and fragment length analysis, we confirmed the presence of the variant in both affected dogs, and its absence in 50 control Labrador Retrievers. Variants in the *NSDHL* gene cause CHILD syndrome in humans, and the bare patches (*Bpa*) and striated (*Str*) phenotypes in mice. Taken together, our genetic data and the known role of *NSDHL* in X-linked skin disorders strongly suggest that the identified structural variant in the *NSDHL* gene is causative for the phenotype in the two affected dogs.

A typical clinical sign of X-linked dominant or semidominant genodermatoses is the characteristic skin patterning in heterozygous females. The mechanism behind these patterns is the random X-chromosome inactivation (also called lyonization) during early embryonic development. In heterozygous females, cells with the inactivated X-chromosome carrying the pathogenic variant give rise to normal skin, whereas inactivation of the wild-type X-chromosome results in skin lesions. This leads to a visible functional mosaicism with patches of normal or lesioned skin following the lines of Blaschko (Happle 2006; Happle 2016). In humans, examples for functional X-chromosome mosaicism include disorders such as X-linked dominant chondrodysplasia punctate caused by variants in the *EBP* gene (Braverman *et al.* 1999; Derry *et al.* 1999), focal dermal hypoplasia with causal variants in the *PORCN* gene (Grzeschik *et al.* 2007; Wang *et al.* 2007), incontinentia pigmenti caused by impaired NF-kB activation due to variants in *IKBKG* (Smahi *et al.* 2000), and the IFAP syndrome known to be caused by variants in *MBTPS2* encoding a zinc metalloprotease (Oeffner *et al.* 2009). Many of these disorders are lethal in hemizygous males. However, disorders that are sublethal or nonlethal in male patients, such as Menkes disease or X-linked dyskeratosis congenita caused by variants in the *ATP7A* and *DKC1* genes, respectively, also exist (Kaler *et al.* 1994; Heiss *et al.* 1998; Happle 2016).

Skin lesions following Blaschko’s lines in heterozygous females are also known in animals with X-linked heritable phenotypes, for example, incontinentia pigmenti and brindle 1 in horses (Towers *et al.* 2013; Murgiano *et al.* 2016), streaked hairlessness in cattle (Murgiano *et al.* 2015), or X-linked hypohidrotic ectodermal dysplasia in dogs (Casal *et al.* 1997).

A unique patterning of the skin occurs in girls with CHILD syndrome (congenital hemidysplasia with ichthyosiform nevus and limb defects, OMIM #308050; Happle *et al.* 1980). This rare X-linked semidominant disorder is caused by heterozygous genetic variants in the *NSDHL* gene, encoding NAD(P) dependent steroid dehydrogenase-like, also termed sterol-4-alpha-carboxylate 3-dehydrogenase, decarboxylating (EC:1.1.1.170), a 3β-hydroxysteroid dehydrogenase involved in cholesterol biosynthesis (König *et al.* 2000). These variants are lethal in hemizygous males (Happle *et al.* 1980). The mutational spectrum of *NSDHL* in CHILD syndrome is broad, and includes missense variants exchanging conserved amino acids in the encoded protein, as well as deletions, insertions or splice site variants and the complete deletion of the gene. As different variants cause a clinically comparable phenotype, it is suggested that they lead to a loss of function in a critical step within the cholesterol biosynthesis pathway, block the synthesis of cholesterol and lead to an aggregation of toxic intermediates (Bornholdt *et al.* 2005; Kim *et al.* 2005). The exact mechanism causing the CHILD phenotype is, however, not known (Kim *et al.* 2005). Clinically, CHILD syndrome is heterogeneous with involvement of different organs, variation in severity, and different degrees of limb hypoplasia. So far, no clear correlation was found between the nature and location of the genetic variants in the *NSDHL* gene and the severity or the differences in clinical signs (Bornholdt *et al.* 2005).

In the present study, we investigated a female Labrador Retriever and her crossbred daughter, who both showed characteristic skin lesions following Blaschko’s lines. Earlier reports described similar, but not identical histologic lesions in six Rottweiler dogs, one Siberian Husky, and five female Labrador Retrievers of American and Canadian origin (Lewis *et al.* 1998; Scott and Miller 2000; Hargis *et al.* 2013). All dogs were female, but the molecular etiology was not investigated in any of these cases. The aim of the present study was to identify the causative genetic variant for the phenotype in the affected Labrador Retriever of European origin and her crossbred daughter.

## Materials and Methods

### Ethics statement

All animal experiments were performed according to the local regulations. The dogs in this study were examined with the consent of their owners. The study was approved by the “Cantonal Committee for Animal Experiments” (Canton of Bern; permits 75/16 and 38/17).

### Clinical examination

A 7-month-old female crossbred dog was presented for a pruritic generalized scaling dermatitis, offensive odor, and severe lameness. Skin lesions had been observed soon after birth. General physical examination and a thorough dermatological workup, including skin cytology, microscopic examination of the hairs, and multiple deep skin scrapings were performed. Complete blood cell count, serum biochemistry panel, urinalysis, hemostatic profile, thyroid hormones, and thyroid stimulating hormone (TSH) measurement were also conducted. Multiple skin biopsies were obtained for histopathologic evaluation.

According to the owners and the referring veterinarian, the dog’s mother, a purebred Labrador Retriever, had been affected by the same dermatological lesions since she was a puppy, and had been biopsied a few years previously. Moreover, the two male siblings of the female index patient died soon after birth. As those data raised the suspicion of a congenital hereditary disease, ethylenediaminetetraacetic acid (EDTA) anti-coagulated blood samples from both the mother and the daughter were collected for genetic investigations.

### Skin biopsies and histopathological examination

A total of four 6-mm punch biopsies were taken from affected skin of the daughter. Three biopsies were taken at the initial visit from dorsal neck, right lateral elbow, and right tibial region. One biopsy was taken 1 yr later from the dorsal neck. All biopsies were taken under local anesthesia and immediately fixed in 10% buffered formalin, embedded in paraffin, cut as 4 µm sections, and stained with hematoxylin and eosin prior to the histological evaluation. Skin formalin-fixed and paraffin-embedded samples from the mother were retrieved from the laboratory archive for histopathological revision.

### DNA isolation

Genomic DNA was isolated from EDTA blood of the two affected dogs using a Maxwell RSC instrument (Promega). We additionally used DNA from EDTA blood of 29 female and 21 male nonaffected control Labrador Retrievers of European origin that had been stored in the Vetsuisse Biobank, and two female American Labrador Retrievers affected by follicular parakeratosis, which were described earlier (Hargis *et al.* 2013).

### Whole genome resequencing, SNP, and short indel calling

For resequencing of one of the affected dogs, we prepared a PCR-free genomic fragment library with 350 bp insert size, and collected roughly 36× coverage data on an Illumina HiSeq3000 instrument (2 × 150 bp). Read mapping, aligning and variant calling was done as described before (Bauer *et al.* 2017). Briefly, sequence reads were mapped to the dog reference genome CanFam 3.1, and aligned using Burrows-Wheeler Aligner (BWA) version 0.7.5a (Li and Durbin 2009) with default settings. The output SAM file was converted to BAM, and the reads sorted by chromosome using Samtools (Li 2011). PCR duplicates were marked using Picard tools (http://sourceforge.net/projects/picard/). To perform local realignments, and to produce a cleaned BAM file, the Genome Analysis Tool Kit (GATK version 2.4.9, 50) was used. Putative SNVs were identified using GATK HaplotypeCaller in gVCF mode, and subsequently genotyped per-chromosome and genotyped across all samples simultaneously (McKenna *et al.* 2010). Filtering was performed using the variant filtration module of GATK. To predict the functional effects of the called variants, SnpEFF (Cingolani *et al.* 2012) software, together with NCBI annotation release 103 for CanFam 3.1, was used. For variant filtering we used 188 control genomes, which were either publicly available (Bai *et al.* 2015) or produced during other projects of our group. A list of these control genomes is given in Supplemental Material, Table S1.

### Structural variant detection

Functional candidate genes located on the X chromosome were visually inspected for structural variants using the BAM file from the affected dog and the Integrative Genomics Viewer (Robinson *et al.* 2011). The selection of candidate genes was based on known X-linked human genodermatoses (Lemke *et al.* 2014), and included *ATP7A*, *DKC1*, *EBP*, *EDA*, *EFNB1*, *IKBKG*, *MBTPS2*, *NSDHL*, *PORCN*, *SAT1*, and *STS*.

### PCR, fragment length analysis, and Sanger sequencing

To confirm the presence of the large heterozygous deletion in the two affected dogs, and its absence in the nonaffected control dogs, we performed a long-range PCR using the three primers NSDHL_F: TGCCATGAACATCTGGAGAG, NSDHL_R1: ACCCCAAACAACGAATCCT, NSDHL_R2: ACAGCTTCCCCTGCTAAGGT, and SequalPrep long range polymerase (Thermo Fisher). In heterozygous dogs, this resulted in PCR products with sizes of 753 bp for the wildtype and 1166 bp for the deletion allele. The product of the primers NSDHL_F and NSDHL_R1 flanking the deletion on the wild-type allele was too long to be amplified using these PCR primers (15,565 bp). The amplified products were analyzed using a Fragment Analyzer capillary electrophoresis instrument (AATI).

We directly sequenced the PCR products to confirm their identity on an ABI 3730 capillary sequencer (Life Technologies) after treatment with exonuclease I and shrimp alkaline phosphatase. The sequence data were analyzed using Sequencher 5.1 (GeneCodes).

### Gene analysis

We used the CanFam 3.1 reference genome assembly for all analyses. The numbering within the canine *NSDHL* gene corresponds to the transcript with the accession XM_014111859.1, and its predicted translated protein with the accession XP_013967334.1.

### Data availability

An IGV screenshot illustrating the large genomic deletion is shown in Figure S1. Whole genome sequencing data of the affected crossbred dog and control dogs used for private variant filtering are listed in Table S1. Control Labrador Retrievers are listed in Table S2. Whole genome sequence data of the affected dog were deposited at the European Nucleotide Archive (ENA, project accession PRJEB16012, sample accession SAMEA104125075).

## Results

### Qualitative phenotype description

**Figure 1:**
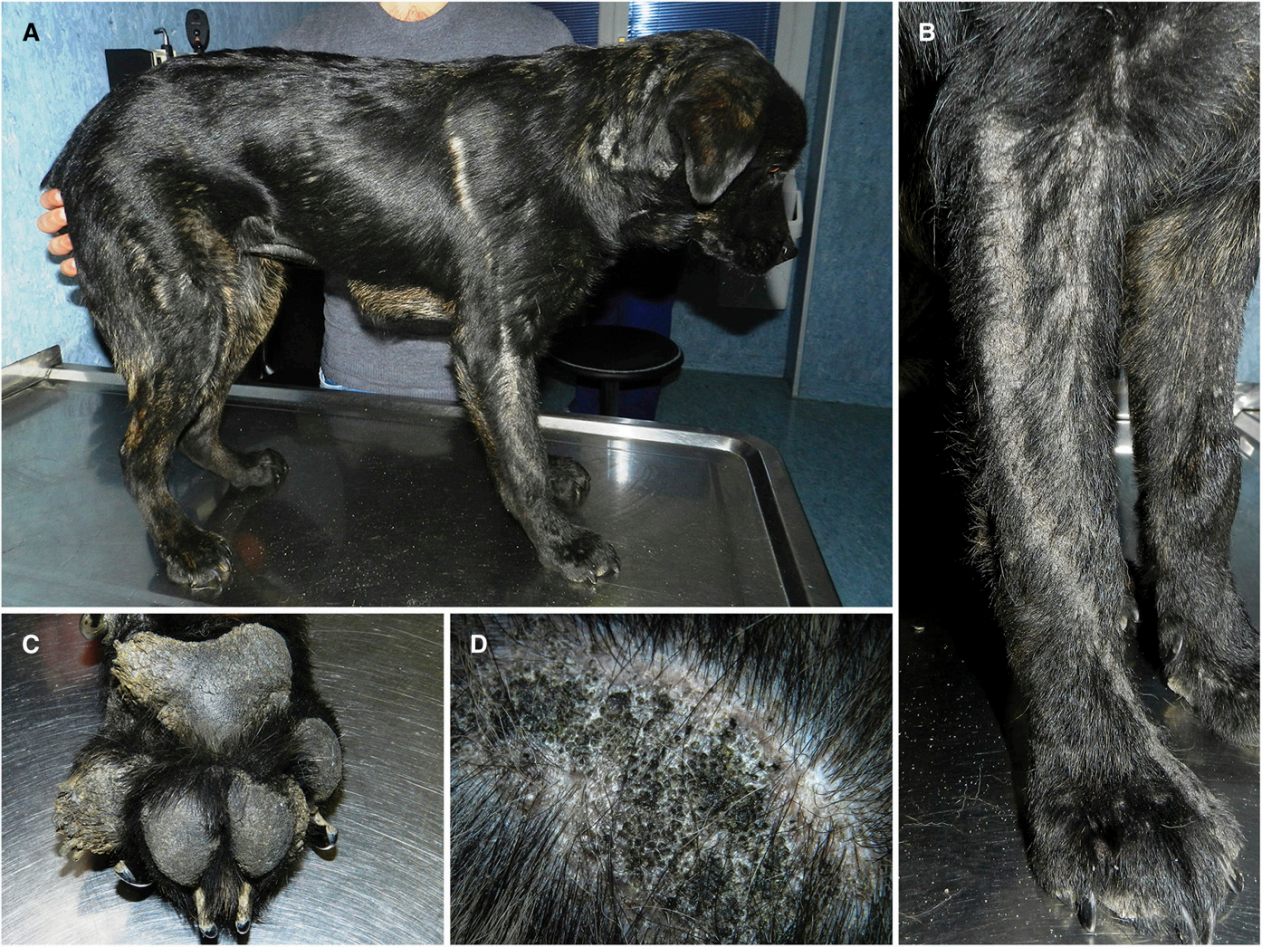
Clinical phenotype. (A) Affected daughter at 7 months of age. (B) Lesions following Blaschko’s lines. (C) Hyperkeratosis of paw pads. (D) Cluster of dilated follicular ostia.

No abnormalities except a stunted growth were found on the affected daughter during general physical examination. Linear hyperplastic and partially alopecic lesions, covered with thick brown scales and clusters of dilated follicular ostia, were the most prominent dermatological features (Figure 1). The lesions were distributed along Blaschko’s lines in a bilateral rather symmetrical fashion, and were more evident on the limbs, the head, the neck, and the dorsal trunk. The abdominal and inguinal skin appeared normal. Frond-like hyperkeratotic lesions at the margin of all the pawpads with occasional horn-like projections were considered the most probable cause of the visible lameness. Cytological examination of the linear hyperplastic lesions revealed the presence of variable numbers of coccoid bacteria, and a large number of *Malassezia* yeasts, which were suspected to substantially contribute to the pruritus and the offensive odor. Results of the blood tests and urinalysis were unremarkable.

### Histopathological examination

**Figure 2:**
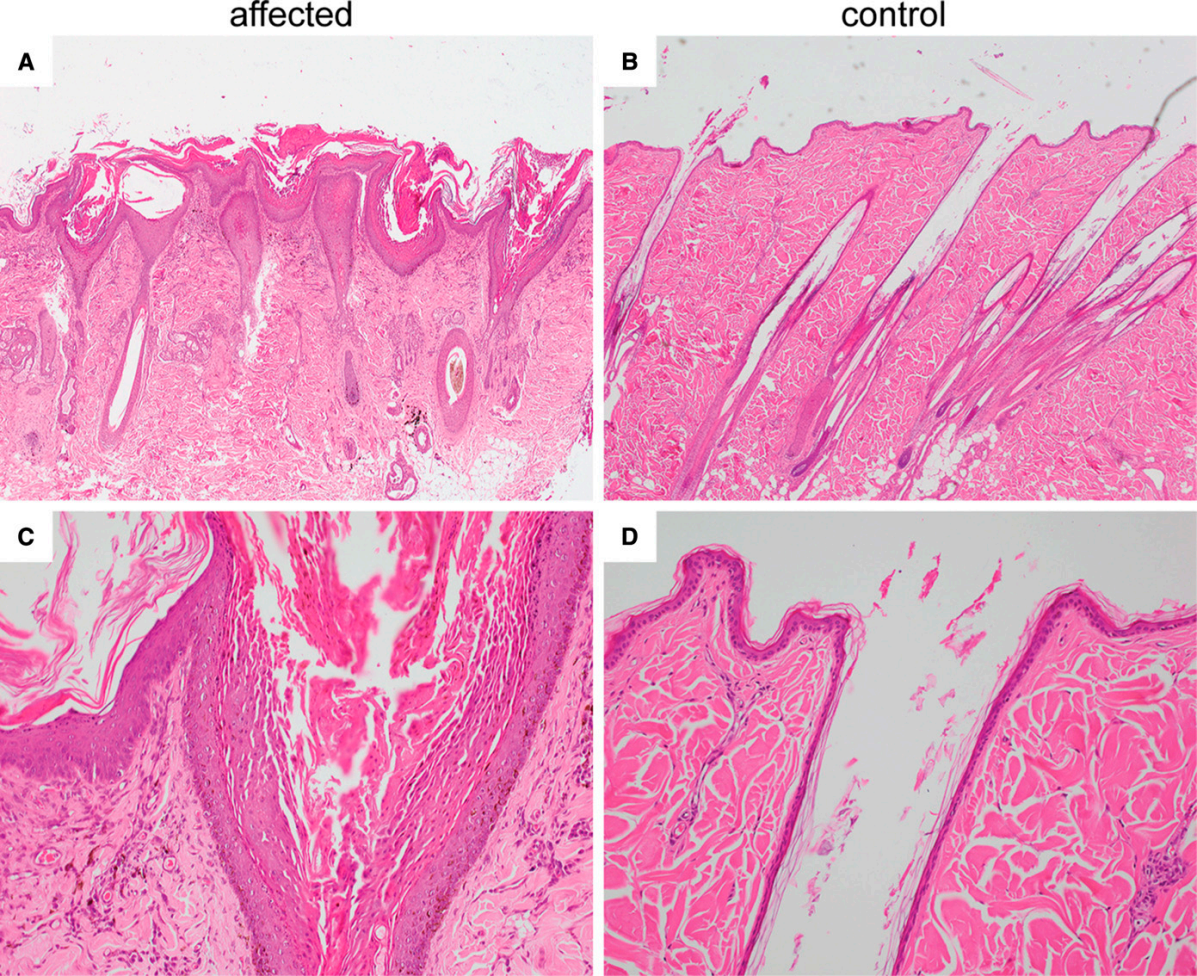
Histopathologic findings in an affected *vs.* control dog. (A) Photomicrograph of a skin biopsy of the affected daughter at 7 months of age depicting a moderately hyperplastic epidermis and severely hyperplastic infundibular epithelium. The infundibuli are filled with densely packed parakeratotic keratin, which is protruding above the epidermal surface. The interfollicular epidermis is covered by moderate to large amounts of laminar to compact, sometimes orthokeratotic, sometimes parakeratotic, keratin. Within the dermis pigmentary, incontinence and a moderate perivascular infiltrate is present. Hematoxylin and Eosin 40×. (B) Skin of a nonaffected dog with normal thickness of the epidermis and infundibular walls. The epidermis is covered by basket-weave orthokeratotic keratin, and the infundibuli are filled with a small amount of orthokeratotic infundibular keratin. Hematoxylin and Eosin 40×. (C, D) Skin sections of the same dogs as in (A) and (B) at higher magnification. Note the severe parakeratotic hyperkeratosis in the infundibulum of the affected dog whereas the neighboring epidermis is covered by orthokeratotic keratin. Hematoxylin and Eosin 200×.

The histopathological findings were identical in all biopsies (Figure 2). Multifocally, the epidermis and the wall of the hair follicular infundibuli were moderately to severely hyperplastic, with abrupt transition to normal skin. Within the hyperplastic area, the infundibular epithelium was covered by thick layers of densely packed parakeratotic keratin, which was distending the infundibuli. The parakeratotic keratin was often protruding above the epidermal surface. The size of the keratohyalin granules within the granular cell layers of the epidermis and the infundibular wall was within the normal range. Within the parakeratotic keratin, multifocally variable numbers of coccoid bacteria were present, and occasionally the lumen of infundibuli contained degenerate neutrophils. Sebaceous glands appeared normal. The interfollicular epidermis was covered by moderate to large amounts of laminar to compact mostly orthokeratotic, but also some parakeratotic keratin. Within the keratin layers of the epidermis, multifocally degenerate neutrophils, nuclear debris, and small numbers of coccoid bacteria were present. Multifocally exocytosis of neutrophils was seen. Within the superficial dermis there was a mild pigmentary incontinence and a moderate perivascular infiltrate composed of neutrophils, mast cells, and fewer lymphocytes.

### Whole genome resequencing

Given the characteristic and comparable skin lesions in the affected dogs, mother, and daughter, which followed Blaschko’s lines, and the perinatal death of the male littermates, we hypothesized that the mode of inheritance was monogenic X-linked semidominant. We resequenced the genome of the affected daughter at 36× coverage, and called SNVs and short indels with respect to the canine reference genome assembly CanFam 3.1. We then searched for heterozygous variants in the genome sequence of the affected dog that were not present in 188 control dogs of different breeds. We found 332 heterozygous variants on the X-chromosome that were absent from the control dogs. However, none of these variants was predicted to be protein-changing variant (Table 1).

**Table 1:** Single nucleotide and small indel variants detected by whole genome resequencing

Filtering Step	Variants*^a^*
Heterozygous variants in whole genome	979,328
Heterozygous variants on X chromosome	25,986
Private heterozygous variants on X chromosome	332
Protein-changing private variants on X chromosome	0

a Only variants that passed the GATK quality thresholds are reported.

### Identification of the causative variant

Given that the automated pipeline did not detect any private protein changing variants on the X-chromosome, we hypothesized that a larger structural variant might be causative for the disorder. Structural variants such as large insertions, deletions, duplications, or inversions would be missed by the applied variant detection software. We therefore selected all 11 known X-chromosomal genes involved in human genodermatoses as functional candidate genes, and visually inspected them for structural variants (Lemke *et al.* 2014). These functional candidate genes were: *ATP7A*, *DKC1*, *EBP*, *EDA*, *EFNB1*, *IKBKG*, *MBTPS2*, *NSDHL*, *PORCN*, *SAT1*, and *STS*.

In the chromosomal region of the *NSDHL* gene, a large structural variant was detected in heterozygous state in the genome of the affected dog. It was a deletion spanning 14,399 bp, including the last three exons of the *NSDHL* gene (Figure S1). The formal variant designation is chrX:120,749,179_120,763,577del14,399. The deletion truncates 192 (53%) of the 361 codons of the wildtype canine reading frame. No structural variants affecting the coding regions of the other candidate genes were detected.

### Fragment length analysis and Sanger sequencing

**Figure 3:**
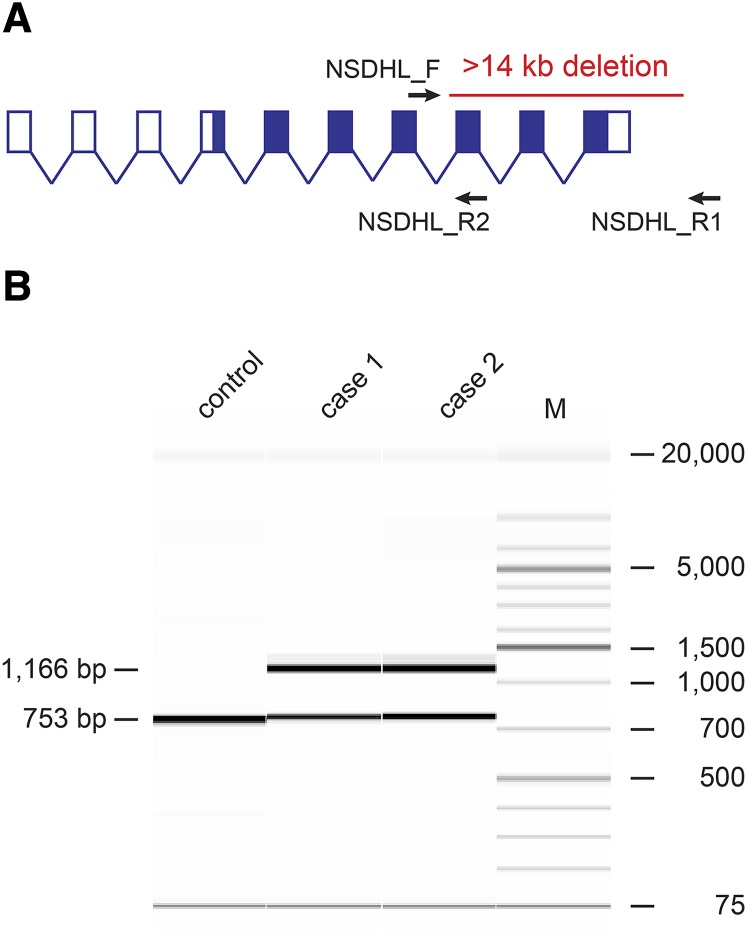
Confirmation of the deletion by PCR. (A) A PCR with the three primers NSDHL_F1, NSDHL_R1 and NSDHL_R2 was performed to genotype cases and controls by fragment length analysis. The exon and intron sizes of the canine *NSDHL* gene are not drawn to scale. The protein-coding region is indicated by solid filling of the exons. Note that the number of 5′-untranslated exons varies between species and transcript isoforms, whereas the seven protein-coding 3′-exons are highly conserved between human, mouse, and dog. (B) A control dog homozygous for the wildtype allele showed only a single band of 753 bp generated by amplification with the primers NSDHL_F and NSDHL_R2. The two cases heterozygous for the deletion showed the wildtype band, and an additional 1166 bp band that resulted from the amplification of NSDHL_F and NSDHL_R1 on the deletion allele. The primers NSDHL_F and NSDHL_R1 did not amplify any specific product on the wildtype allele as their binding sites were >15 kb apart.

To confirm the presence of the deletion in both affected dogs, a PCR approach was chosen. We designed two primers flanking the large deletion as well as one primer inside the deletion, and performed a long-range PCR with these three primers. In the two affected dogs, two PCR products resulted, which were consistent with the expected fragment sizes in a heterozygous genotype (Figure 3). In 50 control Labrador Retrievers, only the smaller wildtype band was detected, as expected for dogs not carrying the deletion. In addition, a PCR with only the primers outside the deletion was performed, and the resulting PCR products in both cases were Sanger sequenced. The electropherograms of the variant allele confirmed the 14,399 bp deletion.

## Discussion

In the present study, we identified a large deletion in the *NSDHL* gene in two female dogs whose pedigree and clinical signs suggested an X-linked semidominant disorder. The ∼14 kb deletion included the last three exons of the *NSDHL* gene, and was present in the heterozygous state in the two affected dogs, but absent in 50 nonaffected Labrador Retrievers. The deletion truncated more than half of the open reading frame including the codons for the single transmembrane domain that anchors NSDHL in the ER membrane (Caldas and Herman 2003). Therefore, it seems unlikely that any protein that might be putatively expressed from the mutant allele would be functional.

In humans, the mutational spectrum of *NSDHL* is broad (Bornholdt *et al.* 2005). One of the known human *NSDHL* variants involves a deletion of the three last coding exons, similar to the canine deletion reported in our study. The girl carrying this deletion had CHILD syndrome, with an inflammatory epidermal nevus affecting the left side of the body. She had oligodactyly, with only three fingers on the left hand and one toe on the left foot (Kim *et al.* 2005). As the name of the human disorder suggests, such limb defects were seen in most patients with CHILD syndrome, albeit with varying severity and location (Bornholdt *et al.* 2005).

The phenotype of the dogs was related, but not identical to the clinical signs of CHILD syndrome in humans. The skin lesions in the two affected dogs followed the lines of Blaschko, but, interestingly, they did not show the typical strict lateralization of the inflammatory nevus seen in almost all human cases (Bornholdt *et al.* 2005). However, in human CHILD syndrome, bilateral involvement and very mild phenotypes without limb deformities have also been reported (König *et al.* 2002; Bittar *et al.* 2006).

Variants in the murine *Nsdhl* gene cause the bare patches (*Bpa*) and striated (*Str*) phenotypes in mice. Characteristic for heterozygous *Bpa* females is the hyperkeratotic skin eruption 5–7 d after birth. The lesions leave bare patches arranged in a typical striped pattern after resolving. Mice with this phenotype may also be affected with skeletal dysplasia, cataracts, and microphthalmia, and they are typically smaller than nonaffected littermates. *Str* heterozygous females show a milder phenotype, which is characterized by a striped coat, visible 12–14 d after birth (Liu *et al.* 1999). In both mouse mutants, limb reduction defects and diffuse skin lesions restricted to one side of the body were not seen, which is similar to the dogs described in the present study, but differing from the human phenotype (Caldas *et al.* 2005). In these mouse models, it was also found that the labyrinth layer of the fetal placenta in affected male embryos was always thinner, and fewer fetal vessels were present, possibly leading to death in midgestation (Caldas *et al.* 2005).

Interestingly, the histopathology of the affected dog skin from our study was very similar to the histopathologic findings in six female American Rottweilers and one female Siberian Husky published earlier (Lewis *et al.* 1998; Scott and Miller 2000). Different from the histology described in these dogs, the interfollicular epidermis in our cases was covered with mostly orthokeratotic keratin, and the parakeratosis was mainly restricted to the infundibulum. Clinically, the main differences between the Labrador Retrievers in the present study and the cases described earlier were the severe involvement of the pawpads and the lack of other noncutaneous congenital abnormalities apart from stunted growth.

In addition, follicular parakeratosis has been reported in five Labrador Retrievers (Hargis *et al.* 2013). These five dogs were also all female and showed multifocal crusted papules and plaques, follicular casts, comedones, and hair loss. In contrast to the phenotype in the two cases from our study, no involvement of nonhaired skin was reported. Interestingly, mural folliculitis and apoptotic cells in the hair follicle infundibili, two prominent histological features in the five dogs described by Hargis *et al.* (2013), were not observed in the two cases described here.

We were able to obtain biobanked DNA samples from two of the five Labrador Retrievers with follicular parakeratosis described by Hargis *et al.* (2013). These dogs did not carry the *NSDHL* deletion. Thus, the genetic cause of the phenotype in these dogs remains unknown.

In conclusion, we identified a large deletion in the *NSDHL* gene in a female Labrador Retriever and her daughter, which is most likely causative for the hyperkeratotic lesions in these dogs. The identified variant is most likely the result of a recent *de novo* mutation event and not widely distributed in the dog population.

## Supplementary Material

Supplemental material is available online at www.g3journal.org/lookup/suppl/doi:10.1534/g3.117.1124/-/DC1.
